# Determination of Epimedin B in Rat Plasma and Tissue by LC-MS/MS: Application in Pharmacokinetic and Tissue Distribution Studies

**DOI:** 10.1155/2017/7194075

**Published:** 2017-06-01

**Authors:** Qianru Feng, Shunjun Xu, Jiejing Yu, Shuai Sun, Liu Yang

**Affiliations:** ^1^Guangdong Provincial Hospital of Chinese Medicine, Guangzhou University of Chinese Medicine, Guangzhou 510120, China; ^2^Guangzhou ImVin Pharmaceutical Co., Ltd., Guangzhou 510663, China

## Abstract

A simple, sensitive, and specific liquid chromatography tandem mass-spectrometric method was developed and validated for the determination of epimedin B in rat plasma and tissue samples. After being processed with a protein precipitation method, these samples were separated on an Agilent Eclipse XDB-C_18_ column with an isocratic mobile phase consisting of acetonitrile and 0.1% formic acid aqueous solution (32 : 68, v/v). The calibration curve of epimedin B was linear over the concentration range from 1 to 500 ng/mL in plasma and tissue homogenate. The method was then applied to pharmacokinetic and tissue distribution studies after a single oral administration of Herba Epimedii extract to SD rats. Results showed that epimedin B reached the plasma peak concentration at 0.4 h and the terminal elimination half-life was 1.6 h in rat plasma, and the plasma area under the curve from time zero to infinity (AUC_0–*∞*_) was 14.35 *μ*g/L·h. The concentration distribution of epimedin B in rat tissue was in the following order: liver > ovary > womb > lung > kidney > spleen > heart > brain, indicating that the compound could be widely distributed in rat, and the reproductive system may be the principal target of epimedin B for female rat.

## 1. Introduction

Herba Epimedii (“Yin-yang-huo” in China), a member of Berberidaceae family, has been utilized for medicinal purposes in China for over two thousand years. It is also cultivated and commonly used as a dietary supplement and crude drug in Japan, Korea, and the Mediterranean region. Herba Epimedii has drawn extensive attention because of its estrogen-like activity and preventing osteoporosis [1–3], improvement of sexual function [4, 5], regulation of immune response [6, 7], and putative anticancer activity [8, 9]. This herb medicine contains a class of characteristic isopentenyl flavonoids [10], which are usually thought to be responsible for the above-mentioned pharmacological activities of Herba Epimedii. Of these isopentenyl flavonoids, epimedins A, B, and C and icariin are the most dominant. In fact, many studies have demonstrated that these isopentenyl flavonoids also possess estrogen-like, antiosteoporosis, and immunomodulatory activities [11–15].

Although traditionally Herba Epimedii is orally administrated to treat diseases, there were some reports that these epimedium flavonoids have poor systematic bioavailability. For instance, the absolute bioavailability of epimedin C is less than 1% after oral consumption of pure compound epimedin C or Herba Epimedii extract [16]. The oral bioavailability of epimedin B and icariin is also very poor based on the past reported study [17, 18]. To explain the contradiction, it is necessary to investigate in vivo process after oral administration of the herb, because absorption, distribution, and excretion characteristics of a compound are the critical factors that may affect systematic bioavailability [19]. Some studies have indicated that these epimedium flavonoids exert their pharmacological effects after biotransformation in intestinal tract [18, 20], which could partly explain the poor oral bioavailability of epimedii flavonoids.

Epimedin B is one of the major bioactive components in Herba Epimedii. To date, only one study investigated its kinetic profile [21], and its tissue distribution has never been reported. There was only one research that reported a validated high-performance liquid chromatography (HPLC) method to quantify epimedin B in rat plasma and its application to kinetic study. Using HPLC to detect and quantify compounds in biological samples may have some advantages but, compared to LC-MS/MS, this method has some limitations such as more time and solvent consuming, low selectivity, and lack of analytical sensitivity. In the above-mentioned report, the LLOQ for epimedin B is 50 ng/mL, which may not be sensitive enough to determine epimedin B in different tissue samples. Furthermore, considering that Herba Epimedii is frequently used for women osteoporosis due to its estrogen-like and antiosteoporosis activities [22–25], we designed this experiment on female rats to find out how rats' body handles Herba Epimedii and whether epimedin B can target or accumulate in specific organs, which was essential to full understanding of the health benefits conferred by this compound as well as Herba Epimedii.

The current study therefore established a simple and sensitive LC-MS/MS method to determine epimedin B levels in various biological samples for the first time. The method was validated in terms of linearity, sensitivity, precision, accuracy, recovery, matrix effect, and stability and successfully applied to pharmacokinetic and tissue distribution studies in female healthy Sprague-Dawley rats following a single oral administration of Herba Epimedii extract (containing 15 mg/g epimedin B).

## 2. Experimental

### 2.1. Chemicals and Reagents

**Figure 1:**
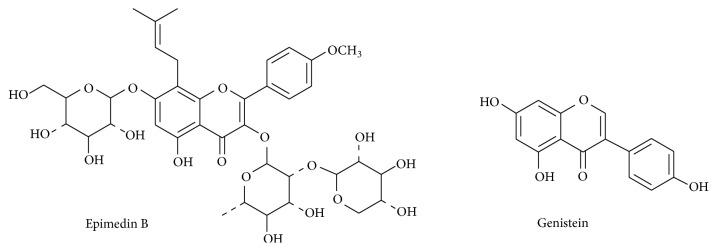
Chemical structure of epimedin B and genistein (internal standard).

Epimedin B (purity 98%) was purchased from Shanghai Ronghe Pharmaceutical Technology Development Co., Ltd. (Shanghai, China). Genistein (IS, purity 98%) was provided by the National Institute for the Control of Pharmaceutical and Biological Products (Beijing, China). The chemical structures of epimedin B and IS are shown in Figure 1. Herba Epimedii standard extract was provided by Guangzhou ImVin Pharmaceutical Co., Ltd. (Guangzhou, China). LC grade methanol and acetonitrile were obtained from Fisher Company (Fisher Scientific, Fairlawn, NJ). HPLC-grade acetic acid was purchased from Tedia Company, Inc. (Fairfield, OH, USA). The deionized water was prepared by a Millipore purification system (Millipore, Milford, MA, USA) and filtered with 0.22 *µ*m membranes.

### 2.2. Instrumentation and Analytical Conditions

LC-MS/MS system consists of a Shimadzu LC-20A HPLC system and an Applied Biosystems Sciex API 4000^+^ (MDS-Sciex, Concord, Canada) equipped with a Turbo Ion Spray. Data acquisition and processing were performed using Analyst 1.6.3 software (Applied Biosystems, Foster City, CA, USA). Chromatographic separation was carried out on an Agilent Eclipse XDB-C_18_ (2.1 × 150 mm, 5 *μ*m), and the column temperature was maintained at 40°C. The mobile phase was composed of acetonitrile-water containing 0.1% formic acid (32 : 68, v/v). The flow rate was set at 0.3 mL/min. The autosampler was set at 4°C and the injection volume was 5 *μ*L.

Mass spectrometer was operated in negative mode for epimedin B and IS. Quantification was performed by multiple reaction monitoring (MRM) mode and the selected monitor ion was* m/z* 867.6 → 645.5 for epimedin B and* m/z* 269.0 → 133.2 for IS. The ESI-MS/MS operation parameters used in this study were as follows: ion spray voltage −4500 V and source temperature (TME) 350°C. Gas 1, Gas 2, curtain gas, and collision gas (nitrogen) were set at 50, 50, 45, and 12 psi, respectively.

### 2.3. Preparation of Stock Solutions, Calibration Standards, and Quality Controls

The stock solutions (1 mg/mL) of epimedin B and IS were prepared with methanol, respectively. The stock solution of epimedin B was diluted with methanol to make a series of working solutions of 5, 50, 500, and 5000 ng/mL. The stock solution of IS was diluted with methanol to make a working solution of 1 *μ*g/mL. All stock and working solutions were kept at −20°C and brought to room temperature before use.

A series of calibration standards were prepared by freshly spiking the appropriate working solution into blank biological matrix to prepare concentrations of 1, 2, 5, 10, 20, 50, 100, 200, and 500 ng/mL for epimedin B and 100 ng/mL for IS. Quality control (QC) samples at three concentration levels (low: 2 ng/mL; medium: 20 ng/mL; high: 400 ng/mL) were independently prepared in the same way. The calibration standards and QC samples were freshly prepared before use.

### 2.4. Sample Preparation

The blank tissue homogenate was prepared by homogenizing drug-free mouse tissue and diluting it with 3 volumes of saline solution. The blank plasma was obtained from drug-free rats.

150 *μ*L of both calibration standard and QC sample spiked with 15 *μ*L of IS was centrifuged through microcentrifuge tubes and the filtrate was dried under a gentle stream of nitrogen. Then 150 *μ*L of drug-free plasma or blank tissue homogenate was added. This was followed by a single step of protein precipitation, by adding 1 mL of methanol and shaking the tubes on a vortex-mixer for 10 min, followed by centrifugation at 14,800 rpm for 10 min. The supernatant consisting of the organic solvent was transferred to fresh tubes and dried under nitrogen. The residue was reconstituted in 150 *μ*L of methanol and centrifuged by a microspin filter tube (0.22 *μ*m nylon, Alltech) at 14,800 rpm for 1 min. A 5 *μ*L aliquot of the solution was injected into the RRLC-MS/MS for analysis.

### 2.5. Calibration Curve

Linearity was assessed by assaying calibration curves on three consecutive days. And the curves were fitted by a weighted (1/*x*^2^) least-squares linear regression method through the measurement of the peak-area ratio of epimedin B to IS. Acceptance criterion for a calibration curve is a correlation coefficient (*r*) of 0.99 or better, and each backcalculated standard concentration must be within 15% deviation from the nominal value except at the lower limit of quantitation (LLOQ), for which maximum acceptable deviation is set at 20%.

### 2.6. Method Validation

#### 2.6.1. Interassay and Intra-Assay Variability

To evaluate the accuracy and precision of the method, QC samples at three concentration levels were analyzed in six replicates on three validation days. The assay accuracy was expressed as (observed concentration/nominal concentration) × 100%. Intra- and interday precision were expressed as relative standard deviation (RSD). The accuracy was required to be within 85–115%, and the two precision values were required not to exceed 15%.

#### 2.6.2. Limit of Quantification

The LLOQ was defined as the lowest concentration on the standard curve at which the standard deviation was within 20% and accuracy was within 100 ± 20%, and it was established using five samples independent of standards. The analyte response at this concentration level should be >10 times the baseline noise.

#### 2.6.3. Matrix Effects (Ionization Efficiency) and Extraction Recovery

To investigate the effect of the sample preparation, the extraction recovery and matrix effect were determined by comparing the peak areas between three types of samples: (1) plasma spiked with known amount of epimedin B before sample preparation; (2) plasma spiked with known amount of epimedin B after sample preparation; (3) pure standard solution of epimedin B. The difference between peak areas of sample 2 and sample 3 reflects the extent of ion suppression. The difference between peak areas of sample 1 and sample 2 reflects the recovery. The accuracy (trueness) of the method was calculated by comparing theoretical and experimentally measured analyte levels.

#### 2.6.4. Stability

The stability of epimedin B in rat plasma and tissue was evaluated using 15 QC samples (six samples at each concentration). The stability was tested under the following conditions: (1) freeze-thaw stability of epimedin B in rat biological sample through three freeze-thaw cycles; (2) short-term stability of epimedin B in rat biological sample at room temperature for 6 h; (3) long-term stability of epimedin B in rat biological sample stored at −80°C for 30 days; (4) postpreparative stability of epimedin B in rat biological sample stored in the autosampler at 4°C for 24 h.

### 2.7. Method Application

In this study, the experimentation on rats obtained approval from an independent ethics committee at Guangdong Provincial Hospital of Chinese Medicine (approval number 2012038). The experiment was performed at an SPF level laboratory, authorized by Guangdong Provincial Government.

18 female Sprague-Dawley (SD) rats (220 ± 20 g) were obtained from Guangdong Medicinal Laboratory Animal Center and kept in an environmentally controlled breeding room for 3 days before the experiments started and fasted for 12 h with free access to water prior to administration. The rats were intragastrically administrated with a single dose of 0.69 g/kg (approximately 15 mg/g epimedin B) of Herba Epimedii extract suspending in water. Animals were euthanized at various time points 0.25, 0.5, 1, 2, 4, and 6 h after dose (*n* = 3 at each time point). Blood was collected via cardiac puncture (about 500 *μ*L) and immediately transferred into heparinized tube. The collected sample was centrifuged at 3000 rpm for 15 min and the supernatant plasma was transferred into another tube and stored at −80°C until analysis. The tissue was immediately removed and rinsed with ice-cold saline to remove extraneous blood and blot-dried and then stored in preweighted and labeled vials at −80°C until use. On the day of analysis, the tissue samples were thawed and weighed to obtain the tissue weight expressed as the difference between the pre- and postvial weights. The tissue samples were homogenized with 3 volumes of saline solution using a tissue homogenizer. Pharmacokinetic parameters were calculated using noncompartmental analysis by DAS Software (ver. 2.0, China State Drug Administration).

## 3. Results and Discussion

### 3.1. Chromatography and Mass Spectrometry Conditions

**Figure 2:**
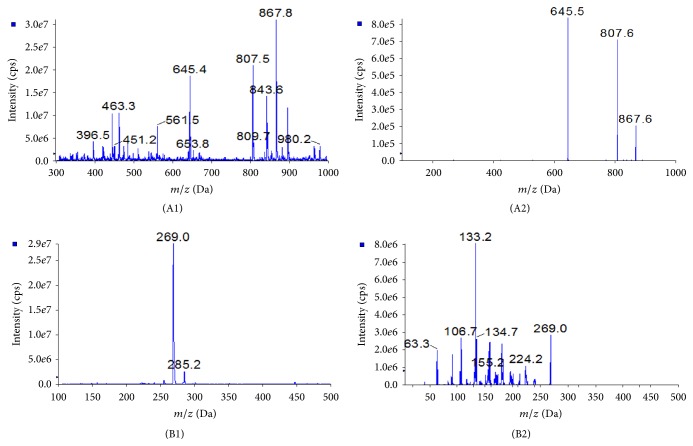
(1) Full-scan mass spectrum of epimedin B (A1) and internal standard (genistein, B1). (2) Product ion spectrum of epimedin B (A2) and internal standard (genistein, B2).

The mass-spectrometric behavior of epimedin B and IS was studied using both positive- and negative-ion ESI. It was found that epimedin B and IS had good responses in negative-ion detection mode with low background noise level. Detection was finally performed in negative-ion mode in this study. The MS/MS spectra of epimedin B and IS were shown in Figure 2.

**Figure 3:**
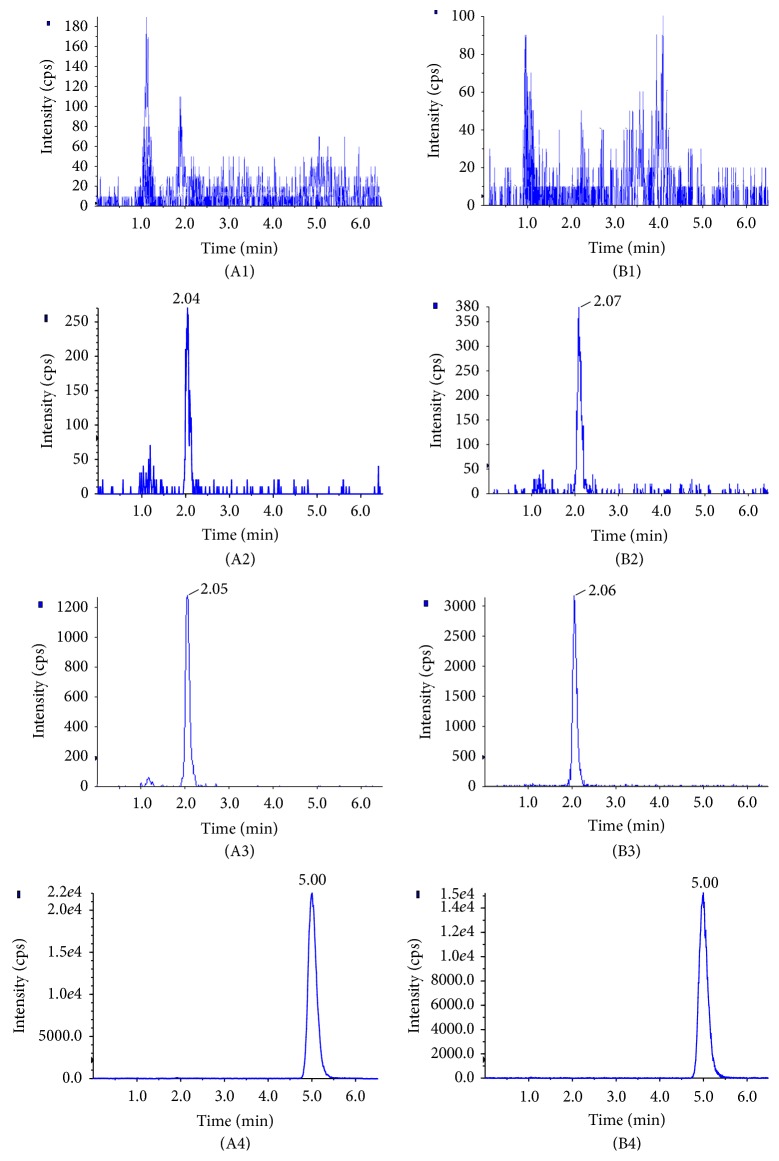
Representative MRM chromatograms: (A1) blank plasma, (A2) blank plasma spiked with epimedin B at LOQ, (A3) rat plasma sample obtained at 0.5 h after administration, (A4) blank plasma spiked with IS, (B1) blank liver, (B2) blank liver spiked with epimedin B at LOQ, (B3) rat liver sample obtained at 0.5 h after administration, and (B4) blank liver spiked with IS.

In order to improve the peak shape and enhance the signal response of analytes, and to reduce the run time, different analytical columns and mobile phase compositions were tried to achieve good resolution and symmetric peak shapes for epimedin B and IS. By comparison with Shiseido capcell pak C_18_ (150 × 2.0 mm i.d. 5 *μ*m) column, Agilent XDB C_18_ (150 × 2.1 mm i.d. 5 *μ*m) column could obtain a better chromatographic behavior and higher signal response for the analytes. Finally, the Agilent XDB C_18_ (150 × 2.1 mm i.d. 5 *μ*m) column was selected for analysis. The modifier of mobile phase of acetonitrile-water binary solvent system was screened from ammonium acetate, acetate acid, and formic acid. As a result, the mobile phase consisting of acetonitrile-water containing 0.1% formic acid with an isocratic elution could improve the symmetry of peak shape and enhance the signal response. The flow rate was also optimized and finally set at 0.3 mL/min. Under the above-mentioned HPLC conditions, the retention time of epimedin B and IS was within 6 min, and no interfering substance was detected at the retention time of analytes in blank rat plasma and tissue samples (Figure 3).

### 3.2. Linearity, Accuracy, Precision, and LLOQ

The assay was found to be linear over the calibration range from 1 to 500 ng/mL for both plasma and tissue homogenate using a weighting scheme of 1/*y*^2^ (*y* = peak-area ratio). The mean linear regression equations were listed in Table 1, with correlations coefficient over 0.99, in which* X* correspond the concentrations and* Y* correspond the peak-area ratios. The LLOQ with an* S*/*N* ratio of >10 was 1 ng/mL, which was sensitive enough for the pharmacokinetic and tissue distribution study of epimedin B.

Inter and Intra-assay variability at three different concentrations, HQC (400 ng/mL), MQC (20 ng/mL), and LQC (2 ng/mL), were determined in plasma and tissue with six replicates on each day for three separate days. The detailed results for inter- and intraday precision and accuracy of epimedin B in biological matrix of rats were summarized in Table 2.

### 3.3. Recovery and Matrix Effects

As shown in Table 3, the extraction recovery of epimedin B was in the range of 81.18–97.60%, which indicated that the recovery of this method was consistent and reproducible and was not concentration-dependent. The suppression of ionization of epimedin B in different biological matrix was distinctly different. For example, the interference of ionization caused by kidney homogenate was significant, whereas the suppression of ionization in brain homogenate was much lower.

### 3.4. Stability

Six replicates of QC at three different concentration levels were used to assess the stability of epimedin B under various conditions. The results are summarized in Table 4, which indicated that epimedin B was stable in autosampler (24 h) at 4°C, bench-top (6 h) at room temperature, and repeated three freeze/thaw cycles and frozen condition at −80°C for 30 days, as the RE values were within ±15% for both the low and high concentrations.

### 3.5. Method Application

**Figure 4:**
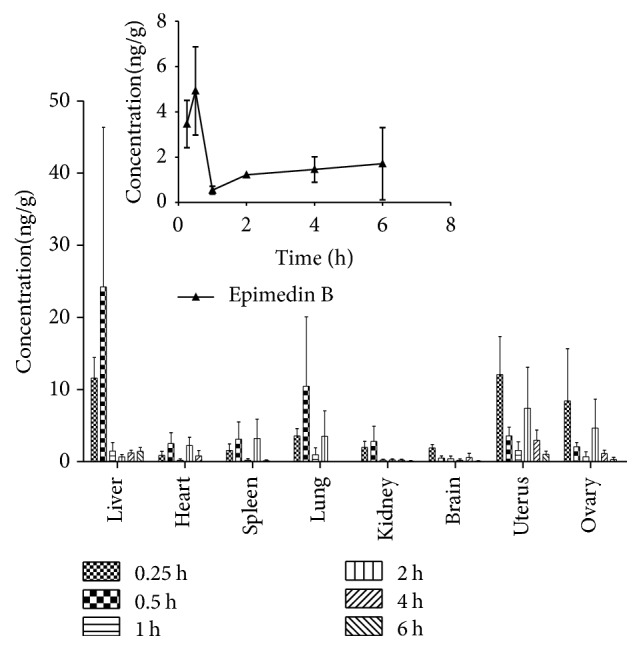
Mean plasma concentration-time profile and tissue distribution of epimedin B in female rats after a single oral dose of Herba Epimedii extract (containing 15 mg/g epimedin B).

The established method was successfully applied for the pharmacokinetic and tissue distribution studies of epimedin B following a single oral dose of 0.69 g/kg of Herba Epimedii extract (approximately 15 mg/g epimedin B) in female SD rat. Plasma and tissue samples were collected at predetermined time points and analyzed using this assay. The assay was proved to be sensitive, reliable, and suitable for batch biological sample analysis. The data showed that epimedin B could reach the plasma peak concentration at 0.4 h after single oral administration to rats. A noncompartmental analysis method yielded a terminal elimination half-life of 1.6 h in plasma, and the plasma area under the curve from zero to time *T* (AUC_0–*t*_) and time infinity (AUC_0–*∞*_) was 9.246 and 14.35 *μ*g/L·h, respectively. As we can see in Figure 4, the curve has an increasing pattern after 2 h. The phenomenon might be caused by enterohepatic circulation. Similarly, many flavonoids also exhibit double-peak or multiple-peak curves after oral administration. All the results demonstrated that epimedin B could be rapidly absorbed in blood and quickly excreted thereafter.

In this study, tissue distribution of epimedin B was also investigated. The data of tissue distribution were expressed as ng/g, calculated by the equation: *C*_*t*_ = *C*_*s*_ × *V*_*s*_/*W*, in which *C*_*t*_, *C*_*s*_, *V*_*s*_, and *W* represent the tissue concentration (ng/g), supernatant concentration, supernatant volume, and the tissue sample weight, respectively. The results showed that epimedin B underwent a rapid and wide distribution in rat tissue within the time course examined. As shown in Figure 4, epimedin B was the least in rat brain, whereas the maximum concentration site was liver, which demonstrated that epimedin B could hardly pass through the blood-brain barrier, and the liver is the highest content distribution site in rats. From Figure 4, the content of epimedin B in female reproductive organs was significantly higher than in other organs at 0.25 h after oral administration of Herba Epimedii extract to rats, which illustrated that epimedin B could be rapidly and largely distributed in female reproductive organs, and those organs might be the target sites of epimedin B. The tissue concentration-time diagram showed that the concentration of epimedin B in various rat tissues would drop to a very low level at 4 h after administration, suggesting no accumulation in tissue and a rapid elimination of this compound.

## 4. Conclusions

A robust, sensitive, and reproducible LC-MS/MS method was established and validated for quantitative analysis of epimedin B in rat biological samples and successfully utilized to evaluate the pharmacokinetic and tissue distribution profiles. The data indicated epimedin B could be quickly absorbed with the plasma peak concentrations at around 0.4 h and the oral bioavailability of epimedin B was very poor after administrating the herbal drug. Tissue distribution studies showed that epimedin B could be rapidly and widely distributed and effectively targeted to female rat reproductive system, like ovary and womb. It is generally thought that Herba Epimedii has estrogen-like activity, which might highly relate to the target distribution characteristic of epimedin B as the main active compound in this Chinese herbal drug extract. To the best of our knowledge, this is the first report for the studies of the in vivo behavior of epimedin B in rats. Results from this work could lay a foundation for further understanding of biological activity of epimedin B and provide useful information for clinical application of the Chinese herbal drug, especially in the treatment of female reproductive system diseases.

## Figures and Tables

**Table 1:** Regression equations of epimedin B in rat plasma and tissue samples (*n* = 3).

Sample	Equation	*R* ^2^	Linear range (ng/mL)
Plasma	*Y* = 0.0049*X* + 0.0012	0.9927	1–500
Liver	*Y* = 0.0045*X* + 0.0011	0.9969	1–500
Heart	*Y* = 0.0045*X* + 0.0005	0.9974	1–500
Spleen	*Y* = 0.0042*X* + 0.0007	0.9941	1–500
Lung	*Y* = 0.0049*X* + 0.0009	0.9957	1–500
Kidney	*Y* = 0.0036*X* + 0.0006	0.9967	1–500
Brain	*Y* = 0.0031*X* + 0.0005	0.9969	1–500
Ovary	*Y* = 0.0045*X* + 0.0011	0.9969	1–500
Womb	*Y* = 0.0045*X* + 0.0011	0.9969	1–500

**Table 2:** Intra- and interday accuracy and precision for the determination of epimedin B in plasma and tissue samples (*n* = 18, 6 replicates per day for 3 days).

Matrix	Conc. ng/mL	Interday RSD (%)	Intraday RSD (%)
Plasma	2	5.528	8.673
	20	12.91	3.314
	400	1.250	3.853

Liver	2	6.264	5.039
	20	1.890	3.058
	400	3.507	1.333

Heart	2	2.216	4.701
	20	1.175	1.755
	400	1.609	1.117

Spleen	2	7.750	6.267
	20	3.824	1.733
	400	6.551	2.624

Lung	2	11.72	4.247
	20	4.767	3.324
	400	7.277	1.714

Kidney	2	14.95	4.529
	20	3.984	2.235
	400	4.209	1.799

Brain	2	10.36	6.442
	20	13.42	2.758
	400	10.25	1.678

Ovary	2	6.264	5.039
	20	1.890	3.058
	400	3.507	1.333

Womb	2	6.264	5.039
	20	1.890	3.058
	400	3.507	1.333

**Table 3:** Matrix effect and extraction recovery for the assay of epimedin B in plasma and tissue samples (*n* = 5).

Matrix	Conc. ng/mL	Matrix effect Mean ± SD (%)	Extraction efficiency Mean ± SD (%)
Plasma	2	61.23 ± 6.029	81.18 ± 5.507
	20	57.59 ± 2.079	82.90 ± 6.316
	400	54.36 ± 1.273	82.55 ± 6.698

Liver	2	68.77 ± 3.563	96.13 ± 4.423
	20	69.55 ± 2.768	89.91 ± 3.414
	400	66.21 ± 2.221	91.38 ± 2.338

Heart	2	61.60 ± 2.829	89.79 ± 3.144
	20	60.22 ± 1.425	92.24 ± 1.202
	400	61.06 ± 1.117	92.30 ± 1.846

Spleen	2	64.60 ± 5.187	88.89 ± 5.246
	20	63.53 ± 2.723	83.55 ± 2.624
	400	64.16 ± 3.018	83.58 ± 0.976

Lung	2	58.72 ± 2.510	92.60 ± 6.441
	20	62.29 ± 1.703	90.24 ± 5.548
	400	62.50 ± 1.793	85.50 ± 1.873

Kidney	2	48.23 ± 3.847	82.85 ± 4.973
	20	42.92 ± 3.564	87.10 ± 4.283
	400	51.48 ± 11.40	83.73 ± 4.550

Brain	2	87.51 ± 3.290	95.80 ± 5.987
	20	90.21 ± 2.125	93.98 ± 2.724
	400	88.48 ± 1.526	97.60 ± 1.525

Ovary	2	68.77 ± 3.563	96.13 ± 4.423
	20	69.55 ± 2.768	89.91 ± 3.414
	400	66.21 ± 2.221	91.38 ± 2.338

Womb	2	68.77 ± 3.563	96.13 ± 4.423
	20	69.55 ± 2.768	89.91 ± 3.414
	400	66.21 ± 2.221	91.38 ± 2.338

**Table 4:** Stability of epimedin B in plasma and tissue samples (*n* = 6).

Matrix	Conc. ng/mL	Mean ± SD			
		Bench-top^a^	Freeze-thaw^b^	Autosampler^c^	Long-term^d^
Plasma	2	92.48 ± 5.190	108.7 ± 1.506	98.70 ± 8.469	94.34 ± 10.42
	20	105.7 ± 8.331	107.8 ± 2.387	102.2 ± 2.077	98.20 ± 1.829
	400	98.74 ± 5.406	94.90 ± 1.238	96.64 ± 4.406	94.44 ± 4.633

Liver	2	107.2 ± 6.700	93.18 ± 4.931	111.6 ± 4.615	103.8 ± 8.422
	20	99.46 ± 2.219	98.78 ± 2.022	111.6 ± 2.302	98.50 ± 2.371
	400	95.90 ± 1.663	101.6 ± 1.342	94.36 ± 1.148	111.2 ± 1.472

Heart	2	109.4 ± 4.450	103.1 ± 4.878	92.12 ± 2.831	99.53 ± 6.960
	20	110.4 ± 0.8944	105.0 ± 1.000	96.72 ± 1.195	101.5 ± 4.622
	400	107.8 ± 1.789	100.1 ± 3.860	91.54 ± 1.144	97.83 ± 5.957

Spleen	2	100.0 ± 4.621	86.10 ± 2.899	103.16 ± 8.049	98.98 ± 5.335
	20	101.9 ± 1.348	111.0 ± 1.871	97.10 ± 1.891	110.3 ± 1.633
	400	99.84 ± 3.065	108.8 ± 1.095	91.18 ± 1.644	95.32 ± 2.890

Lung	2	93.98 ± 4.664	86.18 ± 2.891	111.6 ± 4.336	87.06 ± 5.065
	20	105.4 ± 4.393	94.06 ± 2.192	109.4 ± 4.879	109.8 ± 3.764
	400	95.54 ± 1.250	94.88 ± 0.672	98.52 ± 0.4764	108.7 ± 2.805

Kidney	2	97.62 ± 3.628	103.5 ± 10.13	98.68 ± 4.247	100.6 ± 7.625
	20	102.9 ± 2.910	99.44 ± 1.297	99.84 ± 2.933	95.78 ± 2.413
	400	92.50 ± 1.405	93.98 ± 1.608	93.62 ± 1.672	107.8 ± 1.329

Brain	2	103.2 ± 5.404	97.98 ± 6.197	92.58 ± 7.499	108.0 ± 4.416
	20	98.82 ± 0.782	103.3 ± 1.751	105.5 ± 2.665	94.30 ± 1.719
	400	95.10 ± 2.348	97.90 ± 3.179	99.62 ± 1.744	106.7 ± 2.805

Ovary	2	107.2 ± 6.700	93.18 ± 4.931	111.6 ± 4.615	103.8 ± 8.422
	20	99.46 ± 2.219	98.78 ± 2.022	111.6 ± 2.302	98.50 ± 2.371
	400	95.90 ± 1.663	101.6 ± 1.342	94.36 ± 1.148	111.2 ± 1.472

Womb	2	107.2 ± 6.700	93.18 ± 4.931	111.6 ± 4.615	103.8 ± 8.422
	20	99.46 ± 2.219	98.78 ± 2.022	111.6 ± 2.302	98.50 ± 2.371
	400	95.90 ± 1.663	101.6 ± 1.342	94.36 ± 1.148	111.2 ± 1.472

^a^At least 6 h at room temperature. ^b^At least 3 freeze-thaw cycles. ^c^At least 24 h at 4°C. ^d^At least 4 weeks at −80 ± 5°C.
